# Case Report: Effect of providing penalty kicking tendencies on goalkeeper's motor behaviour and performance: a case study with an on-field intervention on youth football

**DOI:** 10.3389/fspor.2024.1356340

**Published:** 2024-09-12

**Authors:** Vicente Luis-del Campo, Pedro Tiago Esteves, Leonardo Palma Monteiro, Filipe Luis Martins Casanova

**Affiliations:** ^1^ Faculty of Sports Sciences, University of Extremadura, Cáceres, Spain; ^2^ Sports Department, Instituto Politécnico da Guarda, Guarda, Portugal; ^3^ Sport Physical Activity and Health Research & Innovation Center, Rio Maior, Portugal; ^4^ Football Department, Centro de Investigação em Desporto, Educação Física, Exercício e Saúde (CIDEFES), Lusófona University, Lisbon, Portugal

**Keywords:** probabilistic information, onset movement, penalty saves, goalkeeper, football

## Abstract

The penalty kick is a crucial action in a football match that may determine the final outcome. It features a direct interaction between the shooter and goalkeeper where both search for relevant information as a means to achieve their respective performance goals. A case study, composed of an on-field intervention, was designed to analyze the influence of providing in advance penalty kicking tendencies of the shooters on a youth goalkeeping movement onset and saving performance. Data collection took place over 8 training sessions where a U10 young low-skilled, male goalkeeper and shooters were subjected to a penalty-kick shootout task. In each session, the goalkeeper faced a set of 10 penalty kicks recorded by a high-speed HD camera which allowed to calculate the moment that the shooter hit the ball and the goalkeeper movement onset in the direction of ball trajectory. Results showed that the goalkeeper delayed response initiation in the retention phase, when compared to the baseline and training phases, by moving closer to the foot-to-ball contact time by the penalty shooter. From this study, it can be highlighted that goalkeeping saving actions were adapted to the provision of *a priori* information about shooter kicking tendencies, to sustain a higher performance of the young goalkeeper during the penalty duelling.

## Introduction

Within a football game, penalty shootouts may be highly impactful on the final score. For example, in knock-out games in the UEFA Champions League or FIFA World Championship, penalty shout-outs are decisive in 25% of major tournament matches (1). This importance is strongly connected with the fact that the shooter faces an advantageous scenario with respect to the goalkeeper, as three in every four penalties result in a goal (1, 2), and this advantage is even more evident when the penalty is taken by high-skilled players (3). In this context, there is a growing interest in identifying potential explanatory variables of this football-specific action, both from the perspective of the shooter and goalkeeper (4, 5). To exemplify, some studies dedicated to penalty shooters' performance have been focused on variables such as: (i) the location of their gaze or strategy to adopt during the penalty kick (6, 7), (ii) the approach to the penalty kick with a keeper-independent or keeper-dependent strategy (8), (iii) the use of deceptive movements (9, 10), (iv) the influence of footedness (11), or (v) the ball hitting speed towards the goal (12). From the perspective of the goalkeeper, the interplay between technique and speed to anticipate ball trajectory (13), the visual behaviour displayed around the temporal moment of the penalty kick (14), reaction times and the likelihood of saving a penalty (15) have also been reported by the existing literature as influential factors.

A penalty kick defense is characterised as an interceptive action that demands the goalkeeper to be in the right place at the right time. The goalkeeper tends to get a lower performance than the shooter in this football-specific action since the available time for the goalkeeper to intercept the ball, after foot-to-ball contact by the shooter, is less than needed (16). For example, Dicks et al. (6) suggested that football goalkeepers began their movement to a side of the goal between 50 ms and 350 ms before the shooter hits the ball. In this vein, Morya et al. (17) observed that the shooter's performance improved when the goalkeeper started an interception attempt approximately 400 ms before the shooter hit the ball. Contrary, when the goalkeepers delayed the beginning of the saving movements (i.e., 150 ms before the contact with the ball), their performance decreased. In this line, the goalkeepers should avoid the use of the ball-flight to decide where to dive, even though this specifying information may determine the final direction of the ball to the goal (18), as it could constraint their temporal accuracy (i.e., too late to intercept the ball; see (16)). At the same time, football goalkeepers appear to benefit from picking up late information related with ball trajectory to regulate vertical components of the defending action (18). However, there is a need to verify if this perceptual strategy delay is extensible to different age groups and competitive levels.

Most studies investigating goalkeepers’ performance in penalty kick tasks have been carried out in controlled laboratory environments [e.g., (6, 19–21)]. Although this procedure has made it possible to understand how goalkeepers can adapt to time constraints (e.g., which minimal information affords the goalkeeper to accurately predict the final direction of the ball), these experimental designs have been criticised for decoupling perception and action processes (16). In fact, Dicks et al. (6) found that gaze and movement behaviours of goalkeepers were affected by the level of experimental task constraints (e.g., the goalkeepers fixated earlier and displayed a longer duration at ball location during the *in situ* interception condition compared to the other conditions, and also made more saves than the video simulated conditions). Accordingly, the possibility to expand experimental evidence related to penalty kicks within representative experimental tasks may further elucidate how individuals can search and be guided by relevant information under severe spatio-temporal constraints (22).

Anticipating the outcome of an opponent's action is a relevant skill to achieve fast and accurate responses, helping athletes to sustain high performance levels [for a review; see (23)]. According to Dicks et al. (6), kinematic information has been advocated by previous research as the main source of information for action anticipation (i.e., that information related to the opponent's observable movement kinematics). For example, Shafizadeh and Plat (24) found that novice goalkeepers benefited from receiving cues about the position of the non-kicking foot of the shooter in the anticipation of the direction of penalty kicks, compared to the control group. However, the opponents may disguise actions with the endeavour of masking the true intentions of their movements. In doing so, the athletes provide ambiguous kinematic information to increase the opponent's uncertainty about the observed movement, therefore inducing more incorrect responses. To avoid sustaining their anticipatory behaviour on this less certain information, athletes can rely upon contextual information (25). This source of information refers to advanced visual cues, available at the beginning of the hitting sequence or match scenarios, which are prior to the initiation of the opponent's movement [e.g., the location of teammates, opponents and space, or the strengths and weakness of the opponent; see (26)].

The use of this probabilistic situational information is conceived as a perceptual-cognitive skill that would help athletes generate prior expectations about the opponent's hitting options or actions in each sporting situation (27, 28). Thus, the use of this non-kinematic information would provide an opportunity to maintain sensitivity into action anticipating sport situations featured by severe time constraints (e.g., a goalkeeper trying to intercept a penalty kick). To exemplify, the strategical information on a kicker's prior kicking pattern (e.g., the percentage of prior probabilities in which the ball was kicked to a specific place at the goal in previous matches) is a relevant source of information to enhance anticipation in penalty kicks (29). Expert athletes, as a result of their extensive practice and knowledge, seem to be able to perceive and make better use of this contextual information (30), helping them to start their movements earlier (31) or to be more accurate in their responses (27, 28).

This probabilistic information is referred to, in the current study, as the knowledge of an opponent´s action tendencies and preferences, being classified as non-specific contextual information given the fact that stands as a stable source of information (32). Thus, this type of non-kinematic source of information is interpreted using domain-specific knowledge, enabling athletes to form *a-priori* expectations about the action tendencies, before reliable kinematic information becomes available (33). In this line, Luis-del Campo (34) carried out a meta-analysis on the topic of probabilistic information in sport concluding that the experts anticipated more frequently and with higher accuracy than their low-skilled counterparts based on a better use of this contextual information. Also, expert football players showed better adaptation to changes in opponent action preferences (pass or dribble), compared to their less-skilled counterparts, driven by gaze patterns more focused on some key kinematic relevant areas, and not fixating at the player “off the ball” as in the case of the low skill group (35).

Importantly, knowledge of opponents’ action preferences would have a positive effect on anticipatory behaviours only if this contextual information is congruent with the preferred actions of these opponents/teammates. In contrast, detrimental effects on athletes’ performance can be observed if non-kinematic information is incongruent with these action preferences (36). In this vein, Wang et al. (37) concluded that prior cues affected differently the predictions of opponents’ action outcomes, made by goalkeepers of different skill levels, through the observation of a video penalty kick anticipation task (e.g., the expert group showed a better anticipatory judgement of penalty kicks during incongruent trials compared to the group of novice goalkeepers). Similarly, Murta et al. (21) found that experienced and novice goalkeepers anticipated better the direction of penalty kicks presented in video clips according to a congruent condition (i.e., when prior information matched the outcome of that specific penalty kick) compared to an incongruent condition (i.e., the prior information provided did not match the outcome of that particular penalty kick) or against a condition of absence of information. The impact of deceptive information provided by the penalty takers during penalty situations may also imply a differentiated impact on goalkeepers' performances depending on the dimensionality which the information was observed [e.g., the manipulation of the spatial information would affect more in video-based studies, and the temporal information would be more relevant *in situ* tasks; see (38)].

Nowadays, there is a growing debate about the most suitable talent development pathway in football, from grassroots to professional levels, considering both biopsychosocial and socio-political influences [e.g., what starting point would be the more appropriate for an early engagement perspective: An “early specialisation vs. diversification”; see (39)]. In this line, Almonacid-Fierro et al. (40) conducted a systematic review of the literature about football grassroot concluding that a comprehensive approach based on a deep understanding of the game, knowledge of the play, skills, strategies, decision-making, and technical abilities may greatly contribute to the future development of expert football players. More specifically, Duncan et al. (41) have found that the main factor predicting the football technical skills in grassroots youth football players were the fundamental movement skills by using a machine learning approach.

One of the most prominent frameworks on long-term athletic development was proposed by Lloyd and Oliver in 2012 (42). This model praised the importance of working physical capacities and training structures within sensitive periods. For example, the training process of a 10 year-old male football player should include agility, speed, and sport-specific skill development with a low/moderate level of training structure. This phase is also featured by an emphasis on fundamental movement skills and introduction to power and strength. Thus, football practice is strongly associated to motor and cognitive development in children. For example, a group of children with a chronological age of ∼9–10 years, participating in a football exercise program, improved running, coordination, leg strength, and also performed shorter visual discrimination times, compared with their sedentary peers (43). Similarly, those 7- and 8-year-old boys that attended a football school program, integrated in the physical education curriculum, improved their aerobic endurance, flexibility and speed (44). Further, an analysis of practice activities and instructional behaviours of football coaches disclosed a tendency to use analytical training tasks activities with younger age groups and more playing-based tasks with older age groups (45).

The provision of augmented information by the sport coaches (e.g., feedback, instructions) has been a relevant topic in the design of learning environments (8). From an ecological rationale, verbal instructions would be considered as an instructional constraint on motor learning (46). This constraint would help learners to educate their attention to relevant information sources of the sport environment (47). For example, Ward and Williams (28) found that the skilled football players of different U18 categories selected the best option to perform in different video-projected play sequences, compared to their less-successful counterparts, based on a better use of situational probabilities. Additionally, worth of note is the fact that the body of research related to the penalty-kick task involving youth athletes is quite scarce. Mainly, these studies have addressed kicking kinematics and performance (48), psychological factors (49), among others.

To the best of our knowledge, the only study investigating the effect of *a-priori* information about the opponents’ action preferences, using representative scenarios, on goalkeepers’ anticipatory behaviours and performance in football penalty kicks was developed by Navia et al. (29) and involved experienced goalkeepers. They found a tendency to dive significantly more times to the right side of the ball trajectory when there was 80% chance of kicking the ball to one side of the goal, resulting also in increased goalkeepeŕs anticipation (i.e., the goalkeepers improved their motor behaviour and performance in the penalty kick task when there was a high tendency of the penalty taker to kick to one side of the goal).

Currently, there is a lack of experimental data related with the temporal effects of an intervention program, with particular emphasis on youth goalkeepers. Indeed, no studies have measured the impact of prior information provided by the coach about opponents’ action tendencies on goalkeeper's movement onset and performance. As a result, contextual information related to the action tendencies of opponents is likely to remain a relevant research topic in future (50, 51). Based on these research gaps, we conducted an on-field training intervention, to ascertain potential motor behaviour and performance adaptations of a U10 low-skilled goalkeeper during a representative penalty task as a result of the knowledge of the penalty shooters’ tendencies. Specifically, this case-study aimed to address the influence of the penalty shooters’ kicking tendencies on a youth goalkeeper's onset movement and performance when performed saving penalties on the field of play.

## Methods

### Participant

A youth male goalkeeper aged 10 years-old participated in this case study (Height: 120 cm; Weight: 25 kg). This participant initiated football practice at 6 years old and, by the time data collection was made, he was enrolled in three training sessions per week (1 h per session) plus one official match organised by the Football Federation. Two training sessions were made with his teammates and on the third day he performed a specific-goalkeeper training session with goalkeepers from other U10 teams of the same football club. This last session was conducted by a specific goalkeeping coach. The shooters of the study (*n* = 10) were players of the same team which assured an equivalent age-group and experience level than the goalkeeper. They were right-footed (*n* = 6) and left-footed (*n* = 4). The participants’ sport participation corresponds to Tier 2 (Trained/Developmental) of the Participant Classification Framework proposed by McKay et al. (52).

All participants had normal vision and no medical illnesses or injuries. They were familiar with duelling performances in the penalty box (i.e., the shooters shooting penalty kicks to the goal and the goalkeeper trying to intercept them) during past training and/or competitions. However, the goalkeeper was not subjected to any perceptual skill and/or decision-making training to improve saving performance during the penalty round. The study was carried out according to the guidelines of the University's Ethics Committee and the Declaration of Helsinki. Specifically, this study received approval from the Bioethics and Biosecurity Committee on March 6, 2018 (n°33/2018). The parents of each participant signed a written informed consent, containing the goals and tasks to perform during the intervention but were naïve about the hypothesis of the study.

### Study design

We followed the recommendations for inclusion in N-of-1 studies given by Journal Article Reporting Standards (APA Style JARS). This research approach based on a case-study methodology provides a powerful tool to bridge the science-practice gap (53). An intragroup design with one participant and two types of sessions (A and B) was used. In the type A sessions, the coach did not provide contextual information to the goalkeeper about the direction in which the takers kicked the ball to the goal (i.e., sessions without treatment). In contrast, in the type B sessions, the coach provided verbal information to the goalkeeper about the takers’ kicking tendencies (i.e., treatment sessions). The order of these two types of sessions was counterbalanced as follows: 1A-2A-3B-4B-5B-6B-7A-8A. We designed these two types of training sessions, based on the provision or absence of probabilistic information about penalty kicking tendencies, in order to ascertain which led to better motor behaviour and performance across three different moments in time: baseline, practice and retention.

The study design ensured ecological validity since the participant was required to actively save the penalty kicks in the playing field, as when performing in the game context. The rationale for providing this non-kinematic source of information in this football-specific action is the lack of visuomotor experiences that these novice athletes are exposed to during early phases of their careers. Therefore, this intervention aimed to facilitate the use of *a-priori* expectations about the shooters' action tendencies. In fact, literature praises that youth novice goalkeepers may benefit from receiving augmented feedback from the coach about shooters' tendencies as an instructional constraint to guide their defensive actions during the penalty round and, by this means, led to a performance enhancement in competitive environments (54).

### Procedures

The intervention was composed of eight sessions and lasted for one month, with two sessions per week. Each session included one round of 10 penalty kicks. Therefore, the goalkeeper faced a total of 80 kicks along the intervention, respecting the laws of the game (55). The duration of this intervention was according to the maximum attendance and availability that the participant guaranteed for the experiment and based on the previous intervention developed by Dicks et al. (56) in novice goalkeepers with four training sessions and 80 training trials. Specifically, this penalty round was performed after completing the main part of the training session, and prior to the final stretching activity, according to the availability of the goalkeeper. The number of 10 penalties was agreed between the coach and research team, based on his age and physical load performed during the training session to prevent fatigue and/or injuries. In this penalty round there were 10 teammates of the goalkeeper acting as shooters (i.e., each player shot one penalty by session). They all verbally received initial information from the coach about the direction they had to shoot the penalty towards the goal in each training session. Collectively, the coach and the shooters initially agreed on the direction of their penalty kicks to the goal for each training session, being this information unknown to the goalkeeper. As only two kicks had to be directed to one side of the goal, the second kick to that side should be performed during the 9th (second last) or 10th (last) trial of the round (the first one could randomly appear between the 1st and 8th kick during the training sessions). This procedure prevented the goalkeeper from using the familiar contextual information of the preferred side of the shooters to kick the ball to the goal as a mean to improve his saving performance.

Additionally, in the sessions where there was knowledge about the probability of the kicking tendency (type B sessions), the coach provided the following initial verbal information to the goalkeeper: “Eight of the next 10 penalty kicks will be directed to one side of the goal and two will be kicked to the other side of the goal”. We used a high-level of likelihood (80%) to perform the penalty kicks, as previously made by Navia et al. (29), due to the low-skill level of the participant and his low number of visuomotor experiences performing penalty dueling situations. There was a resting period of ∼45 s between each penalty kick to avoid fatigue in the youth athlete and provide a correct initial position to the next kick. The goalkeeper had to remember this initial instructional constraint to use it in advance during the penalty kicks to improve his task performance. On the other hand, in the session in which there was no provision of information (type A sessions), the goalkeeper had to decide what would be the final direction of the ball direction, without prior instruction from the coach because the shooters were free to kick the ball to either side of the goal. Only in the case that the ball missed the goal or was directed to the wrong side, the trial was repeated at the end of the series. During the penalty round of each training session, no communication was allowed between goalkeeper and shooters, and between goalkeeper and the coaches of the team.

The intervention had the following phases: Phase 1 or baseline that included the two first sessions where the goalkeeper did not receive any information about the kicking tendencies (Sessions 1A and 2A). At the third, fourth, fifth, and sixth sessions (Phase 2 or training phase), knowledge about the probability of occurrence of the penalty kicks was provided to the goalkeeper (Sessions 3B, 4B, 5B, 6B). For the last two sessions (Phase 3 or retention phase), no information was provided (Sessions 7A and 8A). The total duration of the ten penalty kicks in each session was approximately 10 min. All penalties were performed in the team's usual training field. Specifically, penalty kicks were performed on a 7-a-side football field, composed of a playing surface of artificial turf, 65 m long × 45 m wide. The penalty area was formed by two lines drawn at right angles to the goal line, 9 m from the inside of each goalpost. These lines extend 9 m into the field of play and intersect a line parallel to the goal line. The area bounded by these lines and the goal line is the penalty area. A mark (penalty mark) is made in the penalty area 9 m from the midpoint of the line between the goalposts and equidistant between the goalposts. A semicircle with a radius of 6 m from the penalty mark is drawn outside each penalty area.

### Instruments and variables

**Figure 1:**
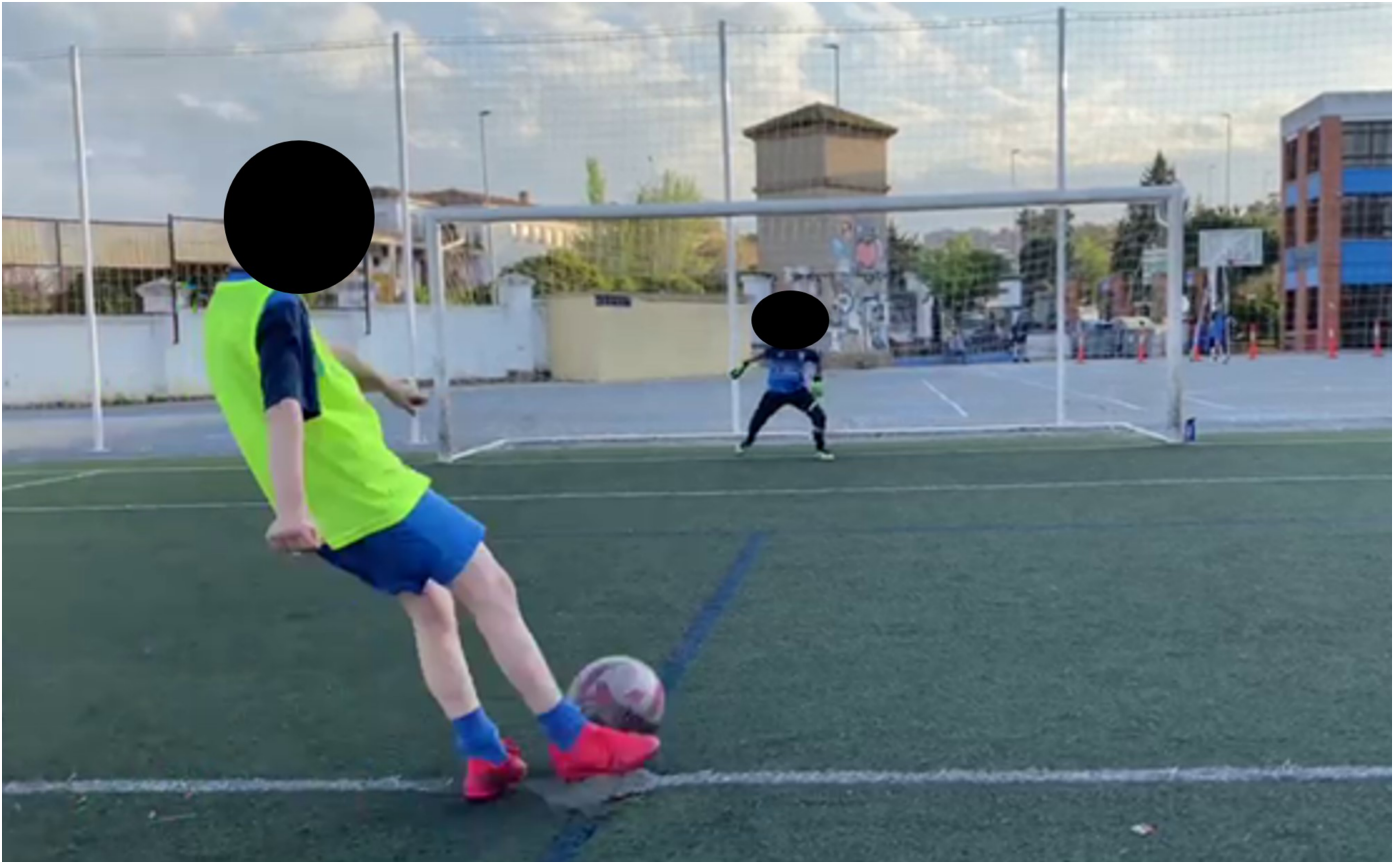
Example of frame in which the goalkeeper starts his stopping movement in relation to the moment in which the shooter hits the ball with the Kinovea software.

We used an iPhone11^TM^ camera, with 4K resolution and frequency of 60 Hz, to record the penalty kicks. This portable device was placed at the back of the semicircle of the large goal area, 3 m away from the players, and slightly towards the kicking leg of the shooter. This spatial location of the mobile on the football field provided a full recording of the penalty's sequences (i.e., a full view of the player's kick and the goalkeeper's response to this kick). The Kinovea program (v.0.10.7) was also used to analyse the onset of the goalkeeper's movement, as well as his performance in defending penalty kicks (see Figure 1).

The independent variable of the present study was the provision of probabilistic information to the goalkeeper concerning the kicking tendencies of the players at different phases of the intervention (*Level 1:* Included the sessions 1A and 2A in which the goalkeeper received no information about the kicking tendencies at the initial baseline; *Level 2*: Included the sessions 3B, 4B, 5B, 6B in which the goalkeeper received information about the probability associated to the kicking direction of the takers at the training phase; *Level 3*: Included the sessions 7A and 8A in which the goalkeeper received no information about the kicking tendencies at the final retention phase).

The onset of the defending movement (ONSET) and the goalkeeper's performance were analysed as dependent variables. Specifically, the ONSET refers to the difference of time (in ms) between the moment that shooter contacts the ball with his foot and the moment that goalkeeper makes the first movement with his foot in the direction of the ball trajectory (57). It has to be noted that the goalkeeper's action may be reactive (i.e., the first movement of the goalkeeper in response to the kick takes place after the player contacted the ball towards the goal) or anticipatory (i.e., the movement of the goalkeeper in the direction of the ball trajectory occurs before the opponent kicks the ball towards the goal). The performance of the goalkeeper (PFM) in the penalty action was also analysed through a scale of five levels that has been previously used to analyse the goalkeeper's performance in this specific-football action (6, 29). The detailed scoring to evaluate saving performance of goalkeeper was: 5 points were assigned when the penalty was saved, 4 points when contacted the ball but failed to save, 3 points when dived to the correct side, 2 points when moved to the correct side, but without diving, 1 point when not moving from the centre of the goal, and 0 points when moved to the incorrect side.

### Statistical analysis

Due to the low number of existing cases (80 penalty kicks), non-parametric statistical analyses were performed to address differences in the dependent variables of the study at the three intervention phases. Firstly, we used descriptive statistics to show mean values and standard deviations for the ONSET and PFM variables. The Friedman test determined if there were differences in the study variables when compared the baseline, intervention, and retention phases. In case of significant differences, the Wilcoxon test was performed to determine differences between pairs of intervention phases. Statistical analysis was performed with the statistical package 15.0 SPSS (Statistical Package for the Social Sciences) (© 2017 SPSS Inc.). Alpha level of <.05 was required for all analyses.

## Results

The Table 1 shows the evolution of ONSET and PFM variables during the eight sessions of the intervention. To highlight an increase of goalkeeper’s performance throughout the four training sessions (3B, 4B, 5B, 6B).

**Table 1:** Descriptive statistics (mean and standard deviation) for the movement onset (ONSET; in ms) and performance of the goalkeeper (PFM; points achieved in a scale from 0 to 5) during each session of the intervention performed in the playing field.

		ONSET	PFM
		M (±SD)	M (±SD)
Baseline	Session1A	−156 ms (118.99)	2.80 (1.75)
	Session2A	−135 ms (107)	2.40 (1.26)
Training	Session3B	−162 ms (112.42)	2.10 (1.19)
	Session4B	−162 ms (171.58)	2.30 (1.05)
	Session5B	−12 ms (139.42)	2.70 (1.41)
	Session6B	−66 ms (145.46)	3.50 (1.50)
Retention	Session7A	9 ms (145.63)	3.60 (.84)
	Session8A	9 ms (163.12)	3.30 (1.16)

**Figure 2:**
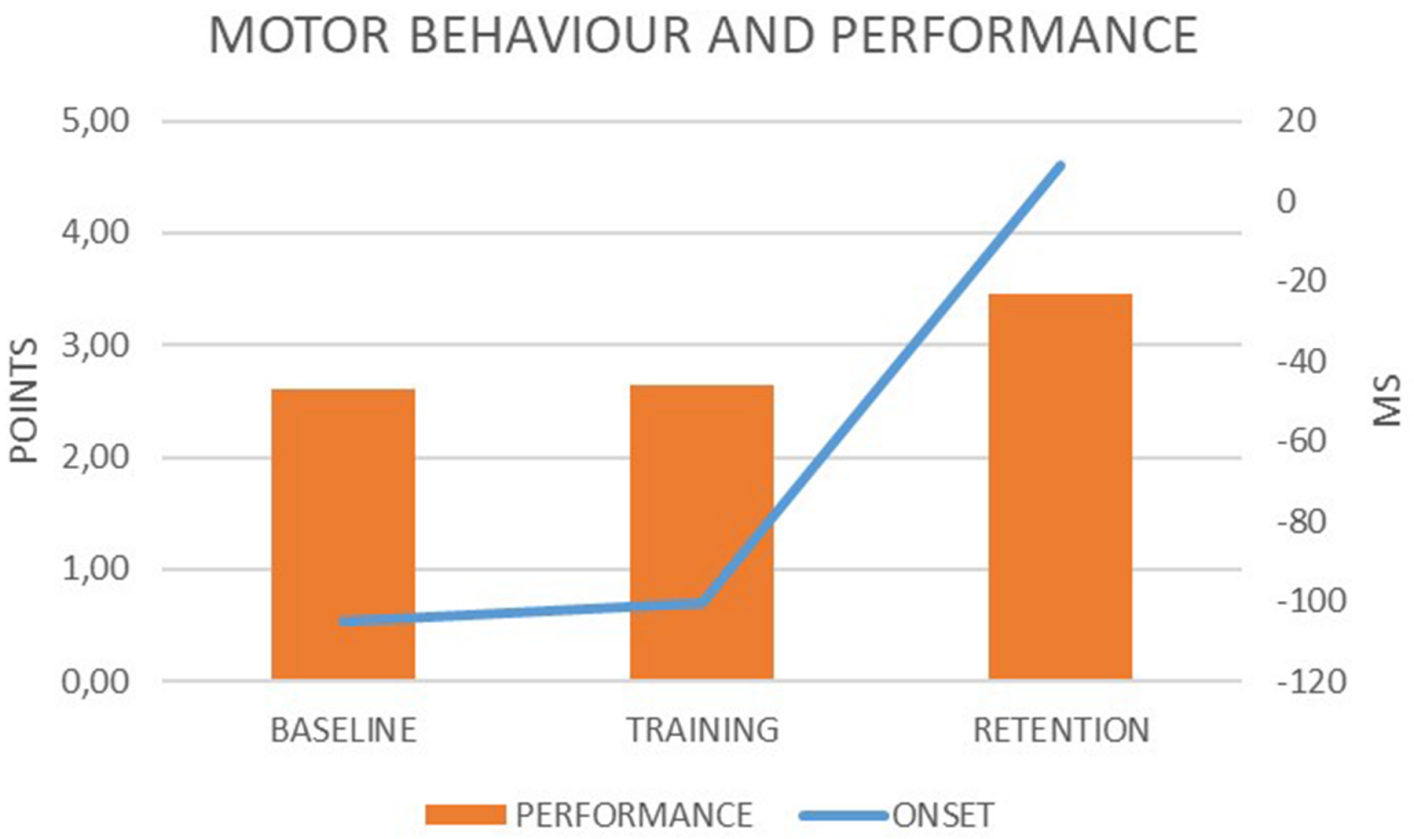
Motor behaviour and performance of the goalkeeper during the penalty task.

The mean values achieved by the participant for the ONSET were −145.50 ms (83.08) at the baseline phase, −100.50 ms (63.36) during the training phase, and 9 ms (70.78) at the retention phase. The goalkeeper showed reactive responses just during the two sessions of the retention phase. For the PFM variable, the mean values were 2.60 (1.10) at the baseline, 2.65 (.85) during the training, and 3.45 (.79) at the retention (see Figure 2).

There were differences in the ONSET values when comparing the three phases of intervention (*X*^2^ = 12.20; *p* < .01). Specifically, these differences were found in the pairwise comparisons between the retention and baseline phases (*Z* = 2.80; *p* < .01), and retention vs. training phases (*Z* = 2.49; *p* < .05). No significant differences were found for the PFM variables between the phases of the intervention (*X*^2^ = 4.15; *p* = .12) although the goalkeeper achieved the highest mean value of PFM at the retention phase.

## Discussion

The aim of the present study was to determine the effects of providing shooters’ penalty tendencies on the movement onset and performance of a youth and novice goalkeeper during an *in situ* penalty-kick task. The results showed that the intervention based on the provision of augmented probabilistic information about shooters’ penalty tendencies caused an adaptation in motor behaviour and performance of the goalkeeper. Specifically, the goalkeeper temporally adapted his defensive responses to initiate them closer to the moment of foot-to-ball contact in the penalty kick for the last part of the intervention program. This delay in the onset movement emerged from Session4B to Session5B. Importantly, this evidence was followed by an increase of performance saving penalty kicks from Session6B to Session8B, with scores higher to 3 points out of 5, although not significant (see Table 1).

We argue that the provision of augmented probabilistic information may have generated an initial expectation about the final direction of the ball towards the goal, helping the youth goalkeeper to better regulate the onset of his interception attempts. These results suggest that the pedagogical strategy used by the coach of not explicitly giving detailed information to the goalkeeper on how and when to move to intercept the ball may have fostered a learning process focused on the available informative variables, both previous and contiguous to the kick (58). In this line, the strategy of providing the goalkeeper with specific probability information of the final direction of ball trajectory became a useful tool to improve task performance, by better adapting his movement patterns to the evolving constraints of the penalty dueling. In this case, the goalkeeper may have better perceived the opportunities for action within the learning environment, under a low level of coach interference because these verbal instructions about shooters’ tendencies were provided just prior to the beginning of the penalty round (and not repeated after each penalty kick). This limited amount of external information prevented interference in the intrinsic feedback system of the goalkeeper during his self-organisation for movement solutions (54).

Previous studies have concluded that coaches’ verbal instructions enhance learning and performance (45), by promoting movement adaptation to the fulfilment of the task aims’ both in competition and training contexts (59, 60). In this vein, explicit verbal feedback by the coach may provide useful assistance to enhance athletes’ exploratory behaviours, involving the use of advanced cues from the shooters’ movements (54). Thus, verbal instructions provided by the coach may have optimized functional couplings of perception and action of the goalkeeper within the specific training environment (61), for instance, helping to better calibrate when temporally should initiate his movement onset according to his action capabilities. Therefore, the provision of specific information based on the probability of occurrence of some events compared to others could help novice athletes to adapt their motor behaviours to the informational demands of the task. In this vein, instructions and external verbal feedback provided during the learning process of the athletes could directly contribute to educate the visual attention of the athletes in training towards the use of more relevant information sources (47, 62, 63). As a result, the goalkeeper significantly delayed movements onset and increased performance scores at the retention phase, compared to the baseline and training phases. Previously, Navia et al. (29) concluded that experienced goalkeepers tended to initiate earlier responses to one side of the goal when prior information was available and that these priors may guide the allocation of visual attention to improve anticipatory performance (50).

Surprisingly, the knowledge of priors about kicking tendencies of the shooters did not lead to an anticipatory performance in the goalkeeper's behaviour. This result is not aligned with previous studies that found earlier responses with high probability conditions (29, 31). Contrary, this contextual information caused a delayed onset of his defensive responses, closer to the temporal moment in which the shooters kicked the ball to the goal, from the middle (Session5) to the end of the training intervention (Session8). This delay on the movement onset was also accompanied by an increase of his saving performance, although not significant. We argue that the goalkeeper learned to initiate later responses during the training sessions by gathering more reliable information about the final direction of the ball to the goal. This additional information would be now associated with the observation of some kinematic cues of the shooters’ movements. According to the temporal values found in Sessions 5B, 6B, 7A and 8A, the goalkeeper may have used the orientation of the non-kicking leg to initiate his responses because it appears ∼160 ms before ball contact. Zhen et al. (64) found that the onset of the goalkeepers’ dives are coordinated with this early kinematic cue because it informs ∼80% about kick direction (65). Therefore, an adaptation in the motor behaviour of goalkeepers with later movement onsets emerged from the integration of the contextual and kinematic information to increase performance scores after the training intervention.

We reasoned that this movement adaptation may have resulted from coaches’ intervention as a learning strategy to pick-up late and relevant information about shooter's action when attempting to shoot at the goal. In particular, contextual information related to the preferred shooting side of the shooters, together with kinematic information about kicking action of the shooters, may have been used by the goalkeeper in the penalty-shooting task. This provided the goalkeeper an opportunity to perceive more reliable and prospective information related to the final direction of the ball (18), such as the position of the hip (66) or the non-kicking foot angle (65), but also to avoid the anticipation cost derived from a flawed prediction response (67). Our findings suggest that the youth goalkeeper may have adapted a perceptual strategy characterised by an attunement to key information variables associated with the kicking action as a result of the manipulation of this informational constraint. However, previous research has shown no differences in visual search behaviours displayed by novice and expert goalkeepers when comparing successful and unsuccessful anticipatory behaviours during penalty kicks, although the experts employed a more efficient visual search strategy characterized by fewer fixations of longer durations than the novices (68). Later, Savelsbergh et al. (69) reported that successful expert goalkeepers fixated longer at the non-kicking leg when compared to their unsuccessful expert counterparts. Navia et al. (29) concluded that the visual behaviours of experienced goalkeepers were affected differently if their actions relied more on situational or body information of the penalty takers. The results found in these studies about goalkeepers’gaze patterns in penalty tasks could be explained by a differentiated perceptual capability to perceive and act on the task (70).

The integration of kinematic and contextual sources of information seems to generate an accurate judgement with the smallest possible response uncertainty (49). For example, Causer et al. (66) found that the hip region was the most relevant cue for a skilled group of goalkeepers (but not for the less-skilled group) to make accurate predictions of penalty kick direction but information from other sources would be needed to make predictions of height. Recently, Huesmann et al. (71) used a qualitative research method to address the anticipation skill and cue utilization of expert handball goalkeepers and goalkeeper coaches when facing backcourt throws. Results of the semi-structured interviews revealed that participants used different kinematic and contextual cues for action anticipation that were available before the game, or before and during the throw. In this vein, these authors encourage that future training programs should integrate both kinematic and contextual cues to enhance goalkeepers’ anticipatory skill.

Some recent neuroscience studies have shown that expert goalkeepers increased their action anticipation performance during a controlled laboratory cue-anticipation task, compared to novice goalkeepers, driven by a proficient modulation of brain activity that emerged from an early attention processing occurred during the integration of prior cues and kinematic information (37). Additionally, Ji et al. (72) have concluded that semi-elite football goalkeepers exhibited a superior process of action anticipation than non-athletes when the priors about the kicking tendencies of the shooters were congruent with subsequent kinematic information of these kicks. In fact, the skilled athletes showed higher level of selective attention toward the characteristics of forthcoming actions during the early phases of kinematic information processing.

Finally, this on-field intervention on youth football enhanced motor behaviour and saving performance of a young football goalkeeper in a short time scale because the total duration of the training lasted 1 month. We argue that the impact of this type of perceptual training would have been higher than the current one if it could be introduced along the sport season (e.g., during the last training before each competition). In doing so, a more prolonged intervention may have revealed a strong learning effect of providing probabilistic information on the motor behaviour and saving performance of the young football goalkeeper. We consider that a longitudinal study would detect developments or changes of the target participant because the variables are measured repeatedly many times and correlated to an individual's current level of performance, but also with a predicted level of performance in the future (73). Irrespective of the time application, this instructional guidance of the coach to the goalkeeper is a key area very pertinent to talent pathway systems that it is related to the talent development because it generates an environment to improve athletes’ potential (74). Indeed, longitudinal research designs provides a predictive value from youth to adult performance level because long interventions in the time enhance the quality of the process of talent development in football (75).

## Conclusion

The contextual information provided by the coach about kicking tendencies during the penalty round influenced the movement initiation of U10 novice goalkeeper participating in this study, as well as his performance obtained in the task. In particular, the participant significantly delayed the onset of his defending movements during the retention phase, compared to the baseline and training phases. Also, this movement adaptation was followed by an increase in performance during this last part of the intervention. Therefore, our findings suggest a potential beneficial effect of providing probabilistic information to a novice goalkeeper about kicking tendencies of the opponents during a penalty kick shooting task. Altogether, this case-study provides novel evidence of the effects of knowing prior cues related to the shooter action outcomes on a goalkeeping penalty kick task.

### Practical applications

The present study may empower coaches to better prepare the training process of youth goalkeepers when performing a penalty kick shootout task by selectively providing useful anticipatory information. From the perspective of the goalkeeper, it seems that the knowledge of a strong kicking preference to one side of the goal during the penalty kicks primed the goalkeeper to delay their saving responses towards that particular direction. This would be useful to achieve a high-performance score if the shooters continue kicking in that direction, but it would be a disadvantage if the shooter does not. Therefore, goalkeepers with lack of specific visuomotor experiences in the penalty task and low skill level would benefit if they observed strong regularities of the penalty shooters' movements that they are congruent with their past behaviour.

From a practical perspective, the exposure to high-probability conditions by the coaches may facilitate novice goalkeepers' performance through a task simplification and guided discovery within the initial skill stage of “*Coordination Training*” (54). In this line, coaches could adopt a scouting strategy for gathering kicking tendencies of the penalty shooters from those teams which later compete. This regular exposure to the penalty dueling during training sessions would facilitate an easier facing of the goalkeeper to this specific action during late competitions.

### Limitations and strengths of the study

The results found in this study can only be applied for the goalkeeper participating in the study. Therefore, it is not possible to make general inferences to larger populations, limiting the generalisability of the results. However, this on-field perceptual training aims to stimulate new interventions dedicated to the manipulation of kicking tendencies of shooters to better prepare the defensive actions of goalkeepers during penalty duelings, irrespective of the age and skill level of goalkeepers. The low number of practice trials used in the current intervention (*n* = 80) may be insufficient for eliciting meaningful improvements in goalkeeper's performance. Public health concerns associated with the Covid-19 pandemic limited a more prolonged intervention with the participant, or the recruitment of more goalkeepers from other clubs. Also, the goalkeeper was relatively familiar with the shots performed by their teammates along the training sessions and this fact could have helped him to identify movement regularities. In future studies, it would be interesting to control this familiarization with the kicking actions performed by the shooters, recruiting other participants from different teams that would ensure unawareness about their kicking tendencies.

The lack of information about the visual search activity of the goalkeeper during the penalty task prevented researchers from having a more complete picture of the motor behaviour and performance achieved by the youth goalkeeper. By monitoring gaze patterns of the goalkeepers, more information could be gained on which kinematic cues were being perceived while performing this interceptive action. For example, Navia, Ruiz et al. (14) found that saving actions are related to short fixations at the area of in front of the ball; instead, a static position during the penalty kick was related with longer fixations towards the area between the ball and the non-kicking leg.

However, we acknowledge that this training intervention was mainly focused on testing the effects of providing information about shooters’ tendencies (i.e., probabilistic information), and not on the analysis of gaze patterns to extract relevant movement cues from the kicks of the shooters (i.e., kinematic information). We designed an experimental landscape to facilitate a self-regulated exploration of the youth goalkeeper's action opportunities during the training sessions, by just providing prior contextual information to guide his defensive movements during the penalty task, with no temporal limitations on movement onsets. In doing so, the youth goalkeeper would avoid that the beginning of his interceptive actions was associated with the late observation of some shooters’ specific kinematic cues, constraining his capacity to temporally respond to the kicks at the previous foot-to-ball contact by the shooter. Within this learning strategy, the coach was deemed as a facilitator of the relations between the youth goalkeeper and the penalty taker (47, 61).

The main strength of this applied study was the collaborative work created between researchers and coaches to enhance the sport performance of a U10 novice athlete, bringing the science-practice gap that usually occurs for the studies in Sport Sciences. We highlight that this training program is the first on-field intervention existing in the literature focused on a youth goalkeeper who would benefit from a coaching assistance to enhance his saving performance during penalty kicks. In this regard, this type of training intervention, focused on grassroots sports, has been overlooked in the scientific community because the main interest have been posited on high-skilled athletes and not in novice participants. This guidance has been based on the role of contextual information related to the action tendencies of shooters, and more specifically on the provision of probabilistic information about the kicking tendencies of shooters. Although the results are referred to this young goalkeeper, we reinforce that the instruments used in this training program could provide portability and usability to the researchers in addressing motor behaviours and saving performance for larger samples of goalkeepers with the same skill level or high-skilled goalkeepers, while guaranteeing reliability recording these variables.

### Future recommendations

Future research addressing the penalty round should include other contextual variables, which have not been considered in the present study, but could influence the motor behaviour and performance of the goalkeepers during this specific football task such as: the footedness of the shooters (i.e., right- vs. left-footed shooters) or the kicking speed (i.e., kicks shooted with high vs. low-velocity). These variables should be also probed with a larger sample of goalkeepers of the same or different ages, skill levels and dissimilar clubs. Thus, it would be interesting to provide knowledge of the execution for each goalkeeper about what is his/her optimal temporal interval at which the saving performance increases. In addition to this, the recordings of gaze patterns of goalkeepers would enhance the comprehension of visual and motor couplings in saving penalties (e.g., what visual target locations are associated with higher or lower saving performance?).

This association between visual and motor behaviour would ensure football coaches a more individualized education of their goalkeepers’ attention during the penalty-shooting task; for instance, by guiding the gaze of their athletes toward specific visual cues that help them to intercept de ball, according to their differentiated action capacities. In doing so, the coaches may manipulate the variability of the informational variables (kinematic cues) and their correlation to the property to perceive (final direction of the ball to the goal) to determinate the advantages by using these variables in each goalkeeper (76).

## Data Availability

The original contributions presented in the study are included in the article/Supplementary Material, further inquiries can be directed to the corresponding author.
